# *Hermetia illucens* and Poultry by-Product Meals as Alternatives to Plant Protein Sources in Gilthead Seabream (*Sparus aurata*) Diet: A Multidisciplinary Study on Fish Gut Status

**DOI:** 10.3390/ani11030677

**Published:** 2021-03-04

**Authors:** Basilio Randazzo, Matteo Zarantoniello, Gloriana Cardinaletti, Roberto Cerri, Elisabetta Giorgini, Alessia Belloni, Michela Contò, Emilio Tibaldi, Ike Olivotto

**Affiliations:** 1 Department of Life and Environmental Sciences, Polytechnic University of Marche, 60131 Ancona, Italy; b.randazzo@staff.univpm.it (B.R.); matteo.zarantoniello@gmail.com (M.Z.); e.giorgini@staff.univpm.it (E.G.); a.belloni@pm.univpm.it (A.B.); 2 Department of Agricultural, Food, Environmental and Animal Science, University of Udine, 33100 Udine, Italy; gloriana.cardinaletti@uniud.it (G.C.); cerri.roberto@spes.uniud.it (R.C.); emilio.tibaldi@uniud.it (E.T.); 3 Council for Agricultural Research and Analysis of Agricultural Economics (CREA), Research Centre for Animal Production and Aquaculture, 00015 Rome, Italy; michela.conto@creagov.onmicrosoft.com

**Keywords:** fish welfare, alternative protein sources, insect meal, gut health

## Abstract

**Simple Summary:**

Sustainability and fish welfare have been receiving increasing attention in the aquaculture sector, with an emphasis on the search for new, sustainable, and healthy aquafeed ingredients. For many years, plant ingredients have been widely used in aquafeed formulation; however, negative side effects on gut welfare have often been reported in several carnivorous fish species. From this perspective, alternative ingredients such as poultry by-products and insect meal are receiving attention due to their low ecological footprint and high nutritional value. In the present study, these two ingredients were used, singly or in combination, to formulate practical diets for gilthead seabream (*Sparus aurata*). After a twelve-week feeding trial, a multidisciplinary laboratory approach including histological, molecular, and spectroscopic techniques was adopted in order to investigate fish physiological responses to the new test diets. The results obtained showed excellent zootechnical performances and ameliorated gut health in fish fed dietary inclusions of poultry by-products and insect meal compared to those fed a vegetable-based diet. In addition, the modulation of nutrient absorption in relation to the ingredients used was highlighted by means of spectroscopic tools. The results obtained demonstrated that poultry by-products and insect meal can be successfully used to replace plant-derived ingredients in diets for gilthead seabream without negatively affecting fish welfare.

**Abstract:**

The attempt to replace marine-derived ingredients for aquafeed formulation with plant-derived ones has met some limitations due to their negative side effects on many fish species. In this context, finding new, sustainable ingredients able to promote fish welfare is currently under exploration. In the present study, poultry by-products and *Hermetia illucens* meal were used to replace the vegetable protein fraction in diets totally deprived of fish meal intended for gilthead seabream (*Sparus aurata*). After a 12-week feeding trial, a multidisciplinary approach including histological, molecular, and spectroscopic techniques was adopted to investigate intestine and liver responses to the different dietary formulations. Regardless of the alternative ingredient used, the reduction in dietary vegetable proteins resulted in a lower incidence of intestine histological alterations and inflammatory responses. In addition, the dietary inclusion of insect meal positively affected the reduction in the molecular inflammatory markers analyzed. Spectroscopic analyses showed that poultry by-product meal improved lipid absorption in the intestine, while insect meal induced increased liver lipid deposition in fish. The results obtained demonstrated that both poultry by-products and *H. illucens* meal can successfully be used to replace plant-derived ingredients in diets for gilthead seabream, promoting healthy aquaculture.

## 1. Introduction

In the last few decades, the exceptional efforts made in finding novel and sustainable ingredients for aquafeed formulation have caused interest in fish health and physiological responses to the new diets [1].

When testing new dietary formulations, an accurate analysis of the organs involved in feed digestion and absorption, immune response, and metabolic processes should be attended. In this regard, the fish gut represents the main target in nutritional challenges, as it plays a primary role in the digestion, absorption, and metabolism of dietary nutrients and local immunity [2,3]; however, metabolic functions are carried out in synergy with other associated organs. The liver, in particular, plays a primary role in the metabolism of dietary nutrients, and its morphology and tissue composition can be deeply influenced by the diet [4,5,6,7].

The complex relation between diet, gut integrity, immune response, nutrient assimilation, and liver morphology and composition has been investigated by means of several laboratory techniques [8,9].

Among these, histological techniques represent the traditional and currently most applied ones used to determine gut responses to dietary challenges based on the analysis of several histo-morphological parameters such as mucosa and sub-mucosa thickness, leucocyte infiltration, and enterocyte lipid vacuolization [10,11]. In addition, dietary challenges may be able to trigger intestine immune response by the activation of a set of pro- and anti-inflammatory-related genes, which may provide information on gut health even in the absence of clear histo-pathological evidence [12,13]. Cytokines such as interleukins and tumor necrosis factor a (*tnf-a*) and inflammation mediators such as nuclear factor kappa-light-chain-enhancer of activated B cells (*NF-kB*) and myeloid differentiation primary response 88 (*MyD88*) represent useful markers of inflammation that are able to provide early information when testing new ingredients in aquafeed formulations [14,15].

To date, besides histological and molecular approaches, target organ responses to dietary challenges were successfully investigated by means of a spectroscopic approach, Fourier Transform Infrared Imaging (FTIR) spectroscopy, which was shown to be able to provide information on gut intestine absorption and liver biochemical composition in different fish species [5,16,17].

Traditionally, vegetable protein sources have represented a readily available, cost-effective, and advantageous alternative ingredient to fish meal (FM) for aquafeed formulation [18,19]. Among the plant ingredients used for aquafeed formulation, soybean meal (SBM) represents the most popular vegetable protein source due to its high protein content, optimal amino acid profile, and digestibility [20,21]. However, SBM is known to exert negative side effects on the gut health and fish welfare of some carnivorous fish species because of its high non-digestible carbohydrate content [22,23] and the presence of anti-nutritional factors (ANFs) [24,25,26]. These negative side effects are particularly known in salmonids [27,28], while studies on Mediterranean euryhaline cultured fish species such as seabream (*Sparus aurata*) are still scarce and often controversial [29].

Though the data of some studies suggest that SBM can be included in seabream diets at relatively high levels [30], the activation of non-specific immune response and intestine inflammation occurrence were reported [31,32]. Similarly, to what has been widely described in salmonids, intestinal morphological changes, including distal intestine leucocyte infiltration and abnormal enterocyte vacuolization, were described in seabream fed a high percentage of SBM [33].

Even if in seabream only a few studies have analyzed the effect of high dietary SBM percentage on immune-related genes in the intestine [32,33], it is well established that SBM ANFs cause gut inflammation by increasing the mRNA levels of pro-inflammatory cytokines in different fish species [13]. In addition, the use of high dietary SBM levels has been shown to affect liver morphology and composition in different fish species [10,34]. Both histological and FTIR analyses showed an increased lipid and glycogen deposition in this organ, even if the results obtained in previous studies are often controversial and seem to be related to the quality of SBM used [33].

For these reasons, finding alternative, sustainable, and cost-effective ingredients that are able to guarantee proper production standards without affecting fish gut health is constantly under exploration [35].

Recently, after the authorization by the EC Regulation No. 56/2013 and No. 893/2017, a range of land-produced feedstuff, such as non-ruminant slaughterhouse by-products (named “processed animal proteins”, PAPs) and insects, have received great attention [36,37].

Among PAPs, poultry by-product meal (PBM) represents a highly available, relatively cheap, sustainable, and valued protein source retaining a high nutrient digestibility and a proper essential amino acid (EAA) profile [38,39]. However, information about PBM’s impact on fish gut health is still limited [40,41].

On the other hand, insect meal has received great attention over the last few years due to its proper amino acidic profile, high protein and mineral content, and low ecological footprint [42]. Moreover, the presence of some biologically active compounds, such as chitin, antimicrobial peptides, and short-medium FAs (such as lauric acid), has been shown to improve gut welfare in different fish species [43,44,45,46].

Recently, PBM [47,48,49,50,51] and insect meal [52,53] were tested as alternative ingredients for seabream diet formulation with promising results in terms of zootechnical parameters, fish health, and fillet quality, while scarce information is so far available about the effects of these ingredients on the fish intestine and liver response. In addition, in the previous experiments, PBM and insect meal were tested as an FM substitute, while the effect of their inclusion on a vegetable-based diet, singly or in combination, is still unknown in gilthead seabream.

A proper combination of these different ingredients may represent an interesting and novel approach for the formulation of a new set of sustainable and possibly highly performing fish diets.

On this basis, the aim of the present study was to investigate gilthead seabream (*S. aurata*), as one of the most important species in Mediterranean aquaculture, by means of a multidisciplinary approach, assessing its growth performance and gut and liver health in response to diets without fish meal, where graded levels of a blend of vegetable protein-rich ingredients (Vp) were replaced by partially defatted *Hermetia illucens* prepupae meal (HM) or PBM as single ingredients and in combination.

## 2. Material and Methods

### 2.1. Ethics

The feeding trial experiment and all the procedures involving animals were carried out in strict accordance with EU legal frameworks relating to the protection of animals used for scientific purposes (Directive 2010/63/EU). It was approved by the Ethics Committee of the University of Udine (Prot. N. 1/2018) and the protocol was authorized by the Italian Ministry of Health (n. 290/2019-PR).

### 2.2. Experimental Diets

Seven test diets were formulated to be grossly iso-proteic (45%), iso-lipidic (20%), and isoenergetic (22 MJ kg^−1^). A diet rich in plant-derived ingredients, named CV, was designed to obtain a 90:10 weight ratio between vegetable and marine protein and a 67:33 weight ratio between vegetable and fish lipid, as calculated from the crude protein and lipid contribution to the whole diet of all marine and plant-based dietary ingredients. Another diet rich in fish meal (CF) was formulated in the opposite way to obtain a 10:90 weight ratio between vegetable and marine protein and a 33:67 weight ratio between vegetable and fish lipid. The remaining diets, named H20, H40, P20, P40, and H10P30, were prepared by replacing graded levels (20 or 40%) of crude protein from the mixture of vegetable protein sources of the CV diet with crude protein from a commercial partially defatted *Hermetia illucens* prepupae meal (HM) and/or poultry by-product meal (PBM) singly or in combination, while maintaining the same 67:33 vegetable to fish lipid ratio as in the CV diet. The proximate analyses and fatty acid profile of the test ingredients are reported as Supplementary Materials (Table S1). Where necessary, the diets were supplemented with essential amino acids to meet the nutrient requirement of *Sparus aurata* [54]. All the diets were manufactured at SPAROS Lda. (Área Empresarial de Marim, Lote C, 8700-221 Olhão, Portugal) by extrusion in two pellet sizes (3 and 5 mm) and stored in a cold room (+4 °C) until they were used. The ingredient composition and proximate analysis of the test diets are shown in Table 1. Feed samples were analyzed in duplicate for dry matter, DM (Association of Official Analytical Chemists—AOAC #950.46), crude protein, CP Nx6.25 (AOAC #976.05), and ash (AOAC #920.153) contents according to AOAC International [55] and for the total lipid content according to Bligh and Dyer, as modified by Burja et al. [56].

### 2.3. Fish Rearing Conditions, Calculation and Sampling

Three-hundred seventy-eight juvenile gilthead seabream (initial mean body weight 48.8 ± 8.8 g) were selected to be uniform in size from a resident stock of 1200 specimens. Fish were divided among 21 cylindrical fiberglass tanks with a capacity of 300 L (18 individuals in each tank) fitted with a device to recover unfed pellets. Tanks were connected to a marine recirculating aquaculture system supplied with sand mechanical and biological filters, a protein skimmer, an ozonator, and a UV lamp (Scubla srl, Remanzacco, Udine, Italia), which ensured the optimal water quality to fish (water temperature, 23.4 ± 0.75 °C; salinity, 31 ± 0.7 g/L; dissolved oxygen, 5.9 ± 0.22 mg/L; pH, 8.0 ± 0.1; TAN < 0.02 mg/L; N-NO2 < 1.0 mg/L). During the feeding trial, fish were kept under a constant day length and intensity (12 h per day at 400 lux) provided by fluorescent light tubes. Fish groups were left to adapt to the culture conditions over two weeks before being randomly assigned in triplicate to the seven dietary treatments. Fish were fed by belt feeders (Scubla srl, Italia), six days a week in two daily meals (8:00 am and 4:00 pm) slightly in excess to satiety over twelve weeks. To this end, each tank was also fitted with an apparatus for recovering uneaten feed pellets shortly after being released by the feeder. Excess satiety was attained by distributing a daily feed amount adjusted to exceed the intake of the previous day to obtain feed residues after each meal. The feed amounts distributed were recorded daily and uneaten feed was recovered, dried, and weighed to estimate the actual feed intake.

At the end of the feeding trial, after a 24 h fasting period (adopted in order to avoid excess in feed gut content, which could interfere with laboratory analysis), all the fish were subjected to stage 3 anesthesia with 80 ppm of MS-222 (PHARMAQ Ltd., Fordingbridge, Hampshire, UK) and individual biometry measurements (total length and body weight) were recorded. Subsequently, three fish per tank (9 fish per dietary treatment) were sacrificed with an overdose (300 ppm) of the same anesthetic and the liver and medium and distal intestine were sampled and properly stored for histological, molecular, and spectroscopic (FTIR) analyses, as described in the following sections.

At the end of feeding trial for each tank-specific growth rate (SGR), the relative feed intake (RFI) and feed conversion ratio (FCR) were calculated as follows:

SGR = 100 × [(ln final body weight−ln initial body weight)/days];

RFI (g/kg/ABW/d) = feed intake per tank/[(initial biomass + final biomass)/2)/days]

were ABW means average body weight;

FCR = feed intake per tank/weight gain per tank.

### 2.4. Histology, Morphometric Analysis, and Histopathological Indexes of Enteritis in Intestine and Evaluation of Fat Fraction in the Liver (PFF)

Samples were prepared according to Randazzo et al. [16]. Briefly, samples (*n* = 9 for each dietary group) from the liver and medium and distal intestine were fixed in Bouin’s solution and stored at 4 °C for 24 h. Samples were then washed and preserved in 70% ethanol solution. After dehydration by graded ethanol solutions, samples were washed with xylene (Bio-Optica, Milan, Italy) and embedded in solid paraffin (Bio-Optica, Milan, Italy). Paraffin blocks were cut with a microtome (Leica RM2125RTS, GmbH, Wetzlar, Germany) and 5 μm sections were stained with Mayer hematoxylin and eosin Y (Sigma-Aldrich, Milan, Italy). Stained sections were examined under a Zeiss Axio Imager.A2 (Zeiss, Oberkochen, Germany) microscope and the images were acquired by means of a combined color digital camera Axiocam 503 (Zeiss, Oberkochen, Germany).

A semi-quantitative evaluation of the distal intestine morphology, based on mucosal fold height, mucosal fold fusion, enterocyte supranuclear vacuoles, and sub-mucosa width, was performed, as previously described by Uran et al. [57] and Zarantoniello et al. [17].

Specifically, for the intestinal fold morphometric evaluation, ten transversal sections of the medium and distal intestine at 200 μm intervals for each sample were analyzed. All the undamaged and non-oblique folds (at least 150 measurements per fish) were measured using the ZEN 2.3 software (Carl Zeiss Microscopy GmbH), and the results were reported as the means of the observations. On the same sections, a semi-quantitative analysis of the histopathological indexes was performed. For the histopathological index score, an arbitrary unit was assigned for each parameter, as described in Panettieri et al. [58].

The sections were analyzed by experienced personnel in two independent blinded evaluations and the score assignment criteria are described in Table 2.

In order to evaluate the percentage of fat fraction (PFF) in the liver, three sections for fish (*n* = 9) for each experimental group at 100 µm intervals were acquired and analyzed by means of the ImageJ software, setting an homogeneous threshold value according to Zarantoniello et al. [43]. Areas that were not evaluable on the sections, such as blood vessels and bile ducts, were not considered. The results were reported as the percentage of the area occupied by fat on the total hepatic parenchyma analyzed on the section.

### 2.5. Fourier Transform Infrared Imaging (FTIRI) Spectroscopy Measurements and Data Analysis

Nine samples of liver and medium and distal intestine were collected for each dietary group and immediately stored at −80 °C. To perform Fourier Transform Infrared Imaging (FTIRI) measurements, for each sample three sections (10 μm thick) were cut using a cryotome 200 μm apart from each other; the sections were deposited without any fixation process onto CaF2 optical windows (1 mm thick, 13 mm in diameter) and then left to air-dry for 30 min [59,60,61].

FTIRI measurements were carried out by means of a Bruker Invenio interferometer coupled with a Hyperion 3000 Vis-IR microscope and equipped with a Focal Plane Array (FPA) detector operating at liquid nitrogen temperature (Bruker Optics, Ettlingen, Germany). On each section, by means of a 15× condenser/objective, specific areas were detected on which the IR maps were acquired in transmission mode in the Mid-InfraRed (MIR) range (4000–800 cm^−1^; spectral resolution 4 cm^−1^; 128 scans). Before each acquisition, the background spectrum was acquired on a clean portion of the CaF2 optical window. All the raw IR maps were pre-processed using the Atmospheric Compensation (to correct for the atmospheric contributions of carbon dioxide and water vapor) and Vector Normalization (applied on the full frequency range to avoid thickness variations) routines (OPUS 7.5 software package, Bruker Optics, Ettlingen, Germany).

#### 2.5.1. Distal and Medium Intestine

The FTIRI analysis was performed on the intestinal folds; each IR map was 164 × 328 µm in size and was composed of 8192 pixel/spectra with 2.56 × 2.56 µm as the spatial resolution.

False color images representing the topographical distribution of lipids, fatty acids, proteins, and glycosylated compounds were created by integrating pre-processed IR maps under the following spectral regions: 3000–2800 cm^−1^ (representative of total lipids, LIP maps), 1700–1480 cm^−1^ (representative of proteins, PRT maps), and 1190–1120 cm^−1^ (representative of glycosylated compounds, COH maps).

From each IR map, 200 IR spectra were extracted in the outermost layer of intestinal folds to evaluate the biochemical composition of the absorbent mucosa of this compartment. On these IR spectra, the following band area ratios were calculated in relation to the total biological mass (TBM) and statistically analyzed: LIP/TBM (ratio between the integrated area of the 3000–2800 cm^−1^ region, representative of total lipids, and TBM, calculated as the sum of the 3000–2800 cm^−1^ and 1780–900 cm^−1^ integrated areas), PRT/TBM (ratio between the integrated area of the 1700–1480 cm^−1^ region, representative of total proteins, and TBM, calculated as described above), and COH/TBM (ratio between the integrated area of the 1190–1120 cm^−1^, representative of glycosylated compounds, and TBM, calculated as described above) (Integration routine, Mode B, OPUS 7.5 software package).

#### 2.5.2. Liver

The analysis was performed on liver sections of all experimental groups, without choosing any region, due to the homogeneity of this tissue. IR maps were 164 × 164 µm in size and were composed of 4096 pixel/spectra with a spatial resolution of 2.56 × 2.56 µm.

False color images representing the topographical distribution of total lipids, unsaturated lipids, fatty acids, proteins, and glycogen were created by integrating pre-processed IR maps under the following spectral regions: 3000–2800 cm^−1^ (representative of total lipids, LIP), 1700–1480 cm^−1^ (representative of proteins, PRT), and 1080–1000 cm^−1^ (representative of glycogen, GLY).

From each IR map, the following band area ratios were calculated and statistically analyzed: LIP/TBM (ratio between the area of the 3000-2800 cm^−1^ region, representative of total lipids, and TBM, calculated as described above), PRT/TBM (ratio between the area of the 1700–1480 cm^−1^ region, representative of total proteins, and TBM, calculated as described above), and GLY/TBM (ratio between the area of the 1080–1000 cm^−1^ region, representative of glycogen, and TBM, calculated as described above).

### 2.6. RNA Extraction and cDNA Synthesis

Samples were prepared according to Olivotto et al. [62,63]. Briefly, the total RNA was extracted from the medium and distal intestine samples (*n* = 9 for each experimental group, approximately 90 mg per sample) using the RNAzol^®^ RT reagent (Sigma-Aldrich^®^, R4533, Milan, Italy) and following the manufacturer’s instructions. The RNA concentration and integrity were analyzed using NanoPhotometer^®^ P-Class (Implen, Munich, Germany) and Gel Red™ (Sigma Aldrich, Milan, Italy) staining of 28S and 18S ribosomal RNA bands on 1% agarose gel, respectively. After extraction, complementary DNA (cDNA) was synthesized from 1 μg of total RNA with the LunaScript RT SuperMix Kit (New England Biolabs, Ipswich, MA, USA), following the manufacturer’s instructions, diluted 1:10 in RNase-DNase-free water, and stored at −20 °C until use. An aliquot of cDNA was used to check the primer pair specificity.

### 2.7. Real Time PCR

The mRNA levels of the selected genes involved in immune response (intestine)—namely, interleukin-1β (*il1b*), interleukin-10 (*il10*), tumor necrosis factor alpha (*tnfa*), nuclear factor kappa-light-chain-enhancer of activated B cells (*nfkb*), myeloid differentiation primary response 88 (*myd88*), and toll-like receptor-1 (*tlr1*)—were assessed. The primers sequences were retrieved from NCBI (http://www.ncbi.nlm.nih.gov/ accessed on 26 January 2021) and are summarized in Table 3 Amplification products were sequenced and the homology was verified. Negative controls revealed no amplification product and no primer-dimer formation was found in the control templates.

PCRs were performed according to Piccinetti et al. [64] and Vargas et al. [5], 2018, in an iQ5 iCycler thermal cycler (Bio-Rad, Hercules, California, USA), and each sample was analyzed via RT-qPCR in triplicate. Reactions were set on a 96-well plate by mixing, for each sample, 1 μL of cDNA diluted 1:20, 5 μL of 2 × concentrated iQ™ Sybr Green (Bio-Rad, CA, USA) as the fluorescent intercalating agent, 0.3 μM of forward primer, and 0.3 μM of reverse primer. The thermal profile for all reactions was 3 min at 95 °C, followed by 45 cycles of 20 s at 95 °C, 20 s at 60 °C, and 20 s at 72 °C. Fluorescent signal were detected at the end of each cycle and melting curve analysis was performed to confirm that only one PCR product was present in these reactions.

For the gene expression relative quantification, beta-actin (*β-actin*) and ribosomal protein S18 (*rps18*) RNA were used as housekeeping genes to standardize the results. Data were analyzed using the iQ5 optical system software version 2.0, including Genex Macro iQ5 Conversion and Genex Macro iQ5 files (all from Bio-Rad). The modification of gene expression was reported with respect to all the groups. Primers were used at a final concentration of 10 pmol μL^−1^.

### 2.8. Statistical Analysis

Growth performance data are expressed as means ± error standard of the means (esm). Data were checked for normal distribution and homogeneity of variance before analysis, and growth parameters were subjected to a one-way analysis of variance (ANOVA). When significant differences were detected, the Tukey’s multiple-comparison test was used to assess the differences among groups. Differences were considered significant when *p* < 0.05. Analyses were carried out using the SPSS-PC release 17.0 (SPSS Inc., Chicago, IL, USA). For the histological and gene expression statistical data analyses, the Graph software package Prism5 (Graph Pad Software, La Jolla, CA, USA ) was used. Histological measurements, IR, and gene expression results were reported as mean ± standard deviation (SD) and were analyzed through an analysis of variance (one-way ANOVA) with a Tukey test for the comparison of the means; the level of significance was set at *p* < 0.05.

## 3. Results

### 3.1. Growth Performances

Fish promptly accepted all the test diets and no mortality occurred throughout the trial. The growth performance of seabream after 12 weeks of the trial is shown in Table 4.

The fish fed diets P20, P40, H40, and H10P30 resulted in a similarly higher growth performance (*p* < 0.05) than those of fish fed diets CV and CF (*p* < 0.05), while fish fed diet H20 showed an intermediate value. Fish fed diet CF resulted in a higher feed consumption when compared to the other dietary treatments (*p* < 0.05), which did not differ from each other.

As a consequence of the reduced growth and increased feed intake, the fish fed on the CF diet exhibited the worst FCR relative to all the other dietary groups (*p* < 0.05), but this was similar to that observed for the fish fed diet CV (*p* > 0.05).

### 3.2. Intestine and Liver Histology

**Figure 1:**
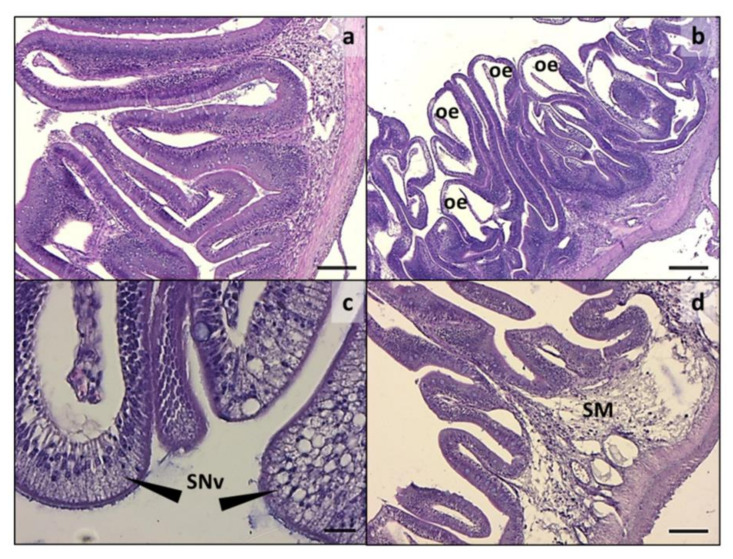
Medium intestine. (**a**) Normal histology from CF; (**b**) histological architecture alteration, including oedema and severe folds fusion degree in CV; (**c**) high magnification showing enterocyte ipervacuolization in CV; (**d**) highly infiltrated and vacuolated submucosa in CV group. oe: oedema; SNv: supranuclear vacuoles; SM: submucosa. Scale: **a**,**d** = 100 µm; **b** = 200 µm; **c** = 20 µm.

**Figure 2:**
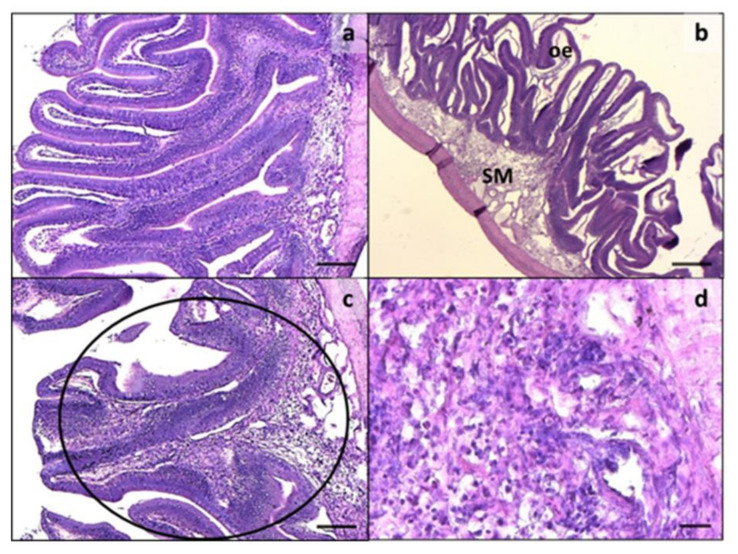
Distal intestine. (**a**) Normal histology from CF; (**b**) histological architecture alterations, including diffuse oedema (oe) and submucosa thickening in CV; (**c**) detail of fold fusion (circle) in CV distal intestine; (**d**) high magnification showing lymphocyte infiltration in CV submucosa. SM: submucosa. Scale: **a**,**c** = 100 µm; **b** = 200 µm; **d** = 20 µm.

Intestine histology showed a variable degree of morphological alteration in both intestine tracts analyzed in all the experimental groups, except for CF (Figure 1a and Figure 2a). A remarkable intensification of the main alterations observed was detected in both the medium and distal intestine from CV group. Particularly, the histological alterations included oedema (Figure 1b and Figure 2b), enterocyte ipervacuolization (Figure 1c), submucosa thickening (Figure 1d), mucosal fold fusion (Figure 2c), and basal inflammatory influx (Figure 2d). The mucosal fold morphometric evaluations and histological index scores are reported in Table 5. Comparing all groups, a significant reduction in fold height was observed in the medium intestine of fish fed diet CV and in the distal intestine of those given diets CV and H20 with respect to the other experimental groups. A significantly higher incidence of fold fusion was also observed in the medium and distal intestine of fish fed diet CV compared to the other groups.

Enterocyte supranuclear vacuolization was significantly increased in the medium intestine of fish given diets H40, P20, P40, H10P30, and CF compared to those fed CV and H20. On the contrary, a higher incidence of supranuclear vacuolization was observed in fish fed diet CV compared to all the other dietary groups. Finally, only diet CV resulted in an inflammatory influx, causing a significant submucosa thickening in the medium and distal intestine (Figure 1d and Figure 2b–d).

**Figure 3:**
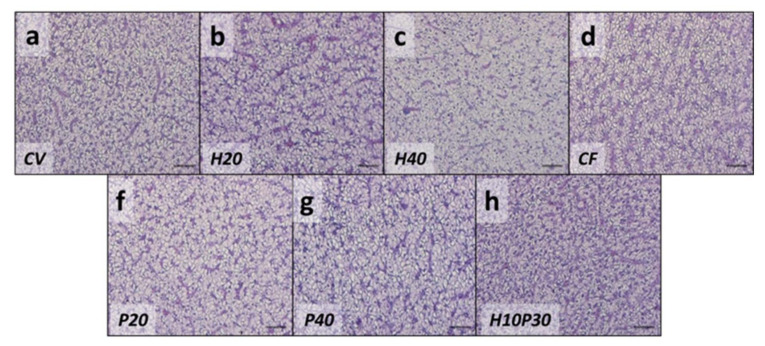
Representative histological images of liver parenchyma from fish fed the experimental diets: (**a**) CV; (**b**) H20; (**c**) H40; (**d**) CF; (**f**) P20; (**g**) P40; (**h**) H10P30. Scale = 100 μm.

Representative images of the liver histological sections from all the experimental groups are shown in Figure 3. All the analyzed samples presented a compact parenchyma with abundant lipid deposition in all the experimental groups. No differences in the percentage of lipid deposition were found by PFF analysis (Table 6)

### 3.3. Gene Expression

**Figure 4:**
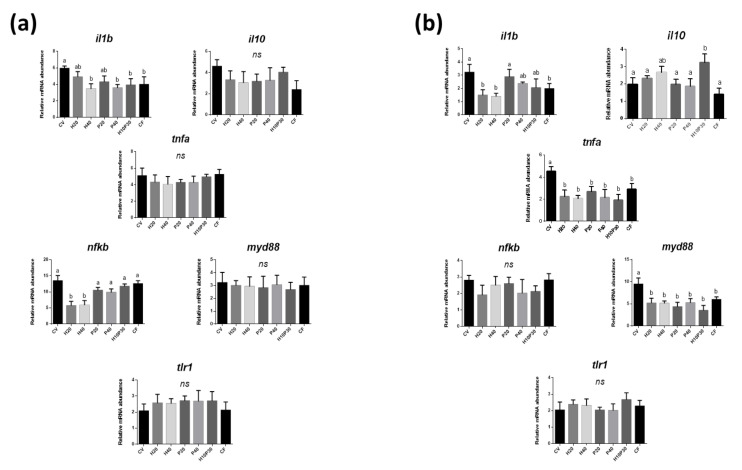
Relative mRNA abundance of genes involved in inflammation and immune response in the medium (**a**) and distal (**b**) intestine. Different letters indicate significant differences among the experimental groups (*p* < 0.05); ns = not significant differences. Values are presented as mean ± SD.

The gene expression analysis performed on medium and distal intestine tissue samples showed a diet-related modulatory effect on inflammatory and immune molecular markers. In the medium intestine (Figure 4a), a significant overexpression of *il1b* was observed in the fish fed diet CV, while a significant downregulation was detected in those given diets H40, P40, and H10P30 compared to the other groups. A similar trend according to the different diets was also observed for the gene expression of *il10*, but it was not significant (*p* > 0.05). In the same intestinal tract, a significant downregulation of *nfkb* was observed in fish fed diets H20 and H40, while no significant differences were observed among the other groups. On the contrary, the gene expression of *tnfa*, *myd88*, and tlr1 was unaffected by dietary treatments (*p* > 0.05).

In the distal intestine (Figure 4b), the magnitude of differences in gene expression due to dietary treatments was more marked. As in the medium tract, il1b was overexpressed in fish fed diet CV compared to CF, with this latter not differing (*p* > 0.05) from the gene expression observed in fish given diets H20 and H40. Fish fed diet P20 did not differ from CV, while all the diets including PBM showed intermediate il1b gene expression between those of fish fed the control diets. In fish fed diet H10P30, the il10 gene expression was higher compared to all the other dietary groups except diet H40, which resulted in intermediate values. The gene expression of *tnfa* and *myd88* was similarly downregulated in fish fed CF or all diets including test ingredients (H20, H40, P20, P40, H10P30) when compared to fish fed diet CV (*p* < 0.05). No differences among dietary groups were observed in the *nfkb* and *tlr1* gene expression, similarly to what was observed in the medium intestine.

### 3.4. FTIRI Analysis

Fourier Transform Infrared Imaging (FTIRI) spectroscopy was used to characterize the macromolecular composition of the medium and distal intestinal mucosa and liver parenchyma. Thanks to false color images, the topographical distribution of the macromolecules analyzed in the intestine and liver samples level was analyzed.

#### 3.4.1. Distal and Medium Intestine

**Figure 5:**
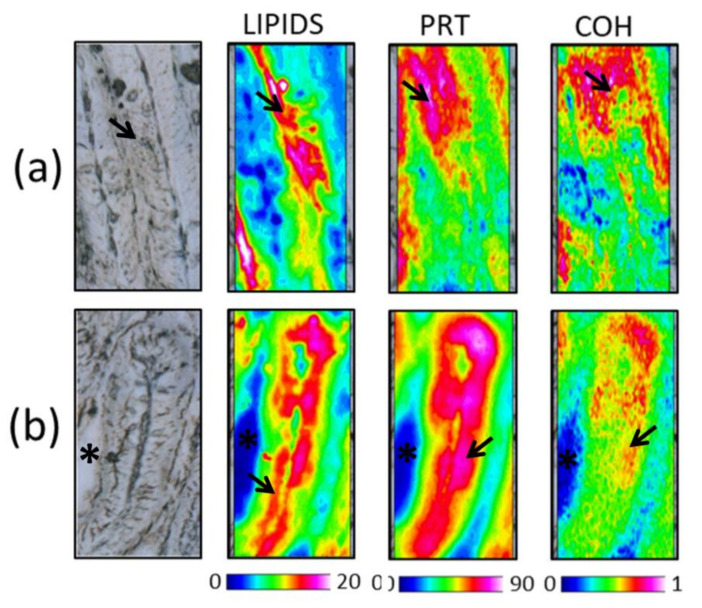
Hyperspectral Imaging analysis of representative sections of medium (**a**) and distal (**b**) intestine of CF dietary groups. False color images (164 × 328 μm) showing the topographical distribution of: lipids (Lip, 0–20 color scale), proteins (PRT, 0–90 color scale), and glycosylated compounds (COH, 0–1 color scale). Black/dark blue colors represent the lowest absorbance values of the infrared radiation, while white/light pink represent the highest ones. Arrows indicate the mucosa epithelial layer; asterisks indicate the intestinal lumen.

The hyperspectral imaging analyses of representative sections of CF medium and distal intestine samples are shown in Figure 5a,b, respectively. False color images showed the topographical distribution of total lipids (LIPIDS maps), proteins (PRT maps), and glycosylated compounds (COH maps). In both intestinal tracts, the main macromolecules considered were predominantly detected at the level of the outermost layer of intestinal folds, which represents the intestine absorbent portion.

**Figure 6:**
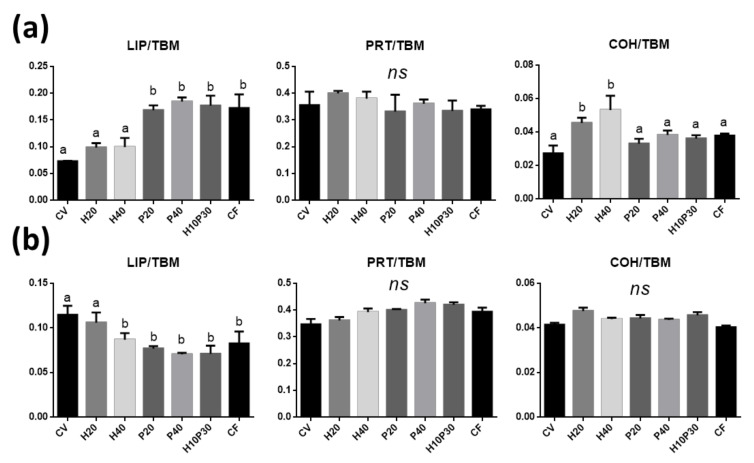
Biochemical composition of medium (**a**) and distal (**b**) intestine mucosa. Statistical analysis of the following band area ratios: LIP/TBM (relative amount of total lipids), PRT/TBM (relative amount of total proteins), and COH/TBM (relative amount of glycosylated compounds).

To evaluate changes in the biochemical composition of the absorbent portion of intestinal mucosa in relation to the different diets, the following band area ratios were analyzed: LIP/TBM (relative amount of total lipids), PRT/TBM (relative amount of proteins), and COH/TBM (relative amount of glycosylated compounds). In the medium intestine (Figure 6a), (i) H20 and H40 samples showed lipid values similar to CV (LIP/TBM, *p* > 0.05), while significantly higher values in P20, P40, H10P30, and CF samples were detected (*p* < 0.05); (ii) no statistically significant differences were found among all groups with regard to proteins (PRT/TBM, *p* > 0.05); (iii) CV, P20, P40, H10P30, and CF exhibited the same amount of glycosylated compounds (COH/TBM, *p* > 0.05), while higher values were observed in H20 and H40 (*p* < 0.05). In the distal intestine (Figure 6b), significantly higher lipid values were found in CV and H20 compared to all the other groups. No statistically significant differences were found among all groups with regard to proteins (PRT/TBM, *p* > 0.05) as well as glycosylated compounds (COH/TBM, *p* > 0.05).

#### 3.4.2. Liver

**Figure 7:**
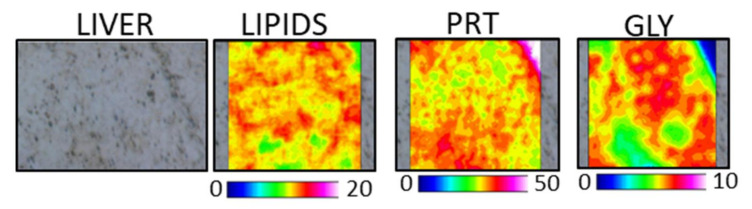
Hyperspectral imaging analysis of a representative section of the liver of the CF dietary group. False color images (164 × 164 μm) representing the topographical distribution of total lipids (LIP, 0–20 color scale), proteins (PRT, 0–50 color scale), and glycogen (GLY, 0–10 color scale). Black/dark blue color represents the lowest absorbance values of infrared radiation, while white/light pink represent the highest ones.

The hyperspectral imaging analysis of a representative section of CF liver is shown in Figure 7; the false color images showed the topographical distribution of total (LIPIDS maps), proteins (PRT maps), and glycogen (GLY maps). As expected based on the structural features of the liver, all the analyzed macromolecules appeared to be homogeneously distributed within the mapped areas; nevertheless, an almost impossible distribution was observed for the total lipids (LIPIDS maps) and glycogen (GLY maps), which was similar to that displayed by proteins (PRT maps).

**Figure 8:**
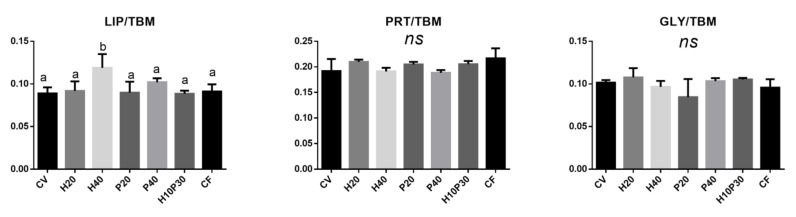
Biochemical composition of liver. Statistical analysis of the following band area ratios: LIP/TBM (relative amount of total lipids), PRT/TBM (relative amount of total proteins), and GLY/TBM (relative amount of glycogen).

Besides the total lipids (LIP/TBM) and proteins (PRT/TBM), the relative amount of glycogen (GLY/TBM) was investigated (Figure 8). With regard to lipids, similar contents were observed in all groups (LIP/TBM, *p* > 0.05) except for H40, which showed significantly higher values compared to the other groups (*p* < 0.05). No statistically significant differences were found among all groups with regard to proteins (PRT/TBM, *p* > 0.05) as well as glycogen (GLY/TBM, *p* > 0.05).

## 4. Discussion

Previous studies demonstrated that gilthead seabream is able to tolerate high levels of dietary FM substitution, either by vegetable or animal protein sources [10,65,66]. However, even if with less extent compared to other carnivorous fish species, moderate adverse side effects on the digestive system of this species were observed when substantial levels of plant-derived ingredients were used in diets [10,31,33,67,68,69].

Accordingly, in the present study the onset of moderate intestine histopathological changes in fish fed a diet in which Vp (vegetable protein-rich ingredients) represented the major protein source (CV) enforces the evidence that plant-derived ingredients represent only a partial alternative to totally replace FM in diets for gilthead seabream. On the contrary, reducing dietary Vp resulted in improved histological gut conditions, including a reduction in the main intestine morphological changes observed in the CV group.

To date, besides protein-rich plant derivatives, other protein sources such as processed animal proteins (PAPs) and, recently, insect meals have been tested in diets for gilthead seabream [48,70,71,72]. Among PAPs, poultry by-product meal has been studied in previous experiments on juvenile sea bream, where it was found not to be detrimental in terms of growth performance and feed efficiency when included at amounts of up to 25% in diets largely based on fish meal [73]. Even higher levels of dietary inclusion of PBM (36%) have been suggested by Sabbagh et al. [52] for culturing sea bream, where it was found to be able to totally replace dietary FM without adversely affecting growth performance. Additionally, *H. illucens* larval meal has been studied as a novel protein source in diets for gilthead seabream. In juveniles, growth, feed efficiency, and nutrient retention were not reduced when HM was included to replace up to 30% of dietary FM protein [74]. In the present study, PBM and HM inclusion resulted in excellent zootechnical performances, in line with the outcomes of earlier studies, even if the results obtained are not readily comparable, firstly because in previous investigations PBM and HM were tested in diets where FM represented the main protein source, while in the present experiment their inclusion occurred in spite of a Vp in diets nearly deprived of FM. Irrespective of the inclusion level in the diet, both test ingredients resulted in improved growth and feed conversion ratios compared to both control diets. A better outcome with diets including PAPs relative to the CV diet could depend on a better overall amino acid balance and reduced levels of antinutritional factors due to reduced levels of Vp as PBM and/or HM, singly or in association, were included in the diet. On the other hand, improved growth performance and efficiency with diets including alternate protein sources compared to the FM control diet has already been described in gilthead sea bream [67,75]. Moreover, in the present study this could be also partially ascribed to a reduced nutrient and energy digestibility of diet CF compared to the test ones due to its high proportion of fair (low)-quality FM [76].

To the best of our knowledge, little is known about the effects of PBM and HM on gilthead seabream gut physiology. In the present study, replacing Vp with PBM or HM (singly or in combination) resulted in no appreciable negative histopathological change in the liver and digestive tract compared to a FM-based diet (CF), demonstrating the tolerance of gilthead seabream at the levels of dietary inclusion actually investigated.

While histological inspection is widely used to provide a reliable picture of intestine condition, the analysis of a set of molecular markers of inflammation is commonly used to obtain deeper information on gut response to dietary challenges. In particular, cytokines are immune-modulating agents acting as pro-inflammatory mediators (*il1b* and *tnfa*) or playing an important role in the adaptive immunity response, as in the case of *il10* [77]. The upregulation of cytokines and other molecular mediators has been observed as a common response to low FM-based diets in several fish species [78]. In this regard, Estruch et al. [69] demonstrated that a long-term feeding period (22 weeks) with a Vp-based diet led to changes in the inflammatory and immune-related gene expression at the intestinal level in gilthead seabream.

The results of the present trial showed that the activation of inflammatory cascade due to high levels of Vp (SBM in particular) composing the CV diet can be observed in a shorter period (12 weeks), leading to the overexpression of some molecular markers such as *il1b*, *nfkb*, *tnfa*, and *myd88* in particular in the distal intestine.

On the contrary, a general downregulation of inflammatory markers was observed when the Vp dietary content was reduced. Considering that improved gut conditions in the present trial were observed regardless of the alternative ingredient used (replacing Vp), especially in the distal intestine, the results can easily be related to a reduction in dietary ANFs characterizing SBM [79], which represented the major protein source in the Vp blend.

Nevertheless, the molecular results showed a more significant inflammatory marker downregulation in fish fed diets including HM, as highlighted by the *il1b* and *il10* gene expression in the distal intestine of fish fed H20 and H40 diets.

Recent studies have demonstrated that an inclusion of HM up to 21% in low FM diets was able to downregulate inflammatory response in rainbow trout [6]. Moreover, HM has been reported to have beneficial effect on gut physiology in different cultured fish species (as reviewed by Gasco et al. [80]). The beneficial effects on fish gut health have been mainly attributed to the presence of certain HM bio-active compounds, such as chitin and medium-short FAs [17,35,44,62,81], exhibiting immune-stimulating, antimicrobial, and/or anti-inflammatory properties [36,82].

Fish tolerance to chitin (and its derivatives) varies among fish species [83], and no studies are available on the in vivo effects of dietary HM inclusion on gilthead seabream gut health. However, in vitro experiments suggest that chitin can enhance cellular immune activity in leucocytes from fish species, even if the mechanisms involved in immune system modulation remain unclear [84].

Moreover, medium-short FAs, such as lauric acid (highly represented in HM), have been associated with anti-inflammatory and immune-boosting properties [46,85,86], providing a further explanation for the mitigation of the inflammation processes observed in the intestine of fish fed diets H20 and H40. However, other possible roles of the fatty acid composition of the diets besides lauric acid cannot be ruled out. In fact, replacing Veg proteins with HM and PBM possibly resulted in changes in the composition of dietary lipids which went beyond the different proportions between fish and vegetable oils. This could have played a role in the physiological response observed. With all test diets being isolipidic, such putative effects might be related to a different dietary fatty acid composition/profile due to a different lipid contribution of the test ingredients to the overall dietary fatty acid profile. This could have resulted in changes in the n−6/n−3 PUFA ratio in diets including different levels of PBM compared to those including HM with potential effects on the balance between pro- and anti-inflammatory eicosanoids.

Besides analyzing the effects of PBM and HM on gut health, the aim of the present study was to investigate the intestine nutrient absorption and the liver biochemical composition in response to the test diets. In previous studies, FTIR analysis was successfully applied in fish intestine, providing a reliable picture of the nutrient absorption in rainbow trout [6,87].

On this basis in the present study, the hyperspectral results obtained by FTIR analysis detected an improved lipid absorption in the medium intestine in response to a dietary replacement of Vp for HM and PBM reduction. Plant-derived ingredients and SBM in particular are known to lower lipid absorption and retention in fish tissues [88,89,90], thus providing a possible explanation for the improved the lipid absorption observed in the medium intestine when dietary Vp was reduced. Nevertheless, the modulation of lipid absorption here observed in the medium intestine suggested a different role of HM and PBM, with the latter resulting in increased lipid absorption, as observed in groups fed on P20, P40, and H10P30 diets compared to H20 and H40 ones. To some extent, this could depend on the different unsaturation degree of the lipid fraction of the two test ingredients, as it is well known that lipid absorption and digestibility appear negatively correlated to the dietary inclusion level of saturated fatty acids [91], which are notably higher in the lipid fraction of HM compared to PBM.

A different scenario was observed in the distal intestine. Indeed, the higher values of total lipids detected in this tract in fish fed the *CV* diet should not be interpreted as improved lipid absorption, with lipids being mainly absorbed in the proximal intestine [92,93]. Lipid abundance shown by FTIR analysis in this tract of the intestinal mucosa could result from the abnormal enterocyte ipervacuolization observed by histological analysis. Lipid enterocytes vacuolization represents a physiological condition in the fish proximal and medium intestine, while it has been considered as a pathological sign in the distal intestine of different fish species, including gilthead seabream [94,95]. It should be noted that this condition was markedly reduced in fish fed on the CF diet as well as diets including intermediate or high levels of the test ingredients; however, further investigation are needed in this regard. Additionally, the FTIR analysis of the medium intestine showed a dose-dependent increase in glycosylated compounds, representative of carbohydrates, in fish fed graded dietary levels of HM compared to all the other dietary treatments. This could be a result of chitin degradation and absorption, suggesting the ability of gilthead seabream to digest this polysaccharide [96].

In this study, the histological analysis of liver did not show appreciable differences in lipid accumulation, while FTIR revealed a higher lipid deposition in the fish fed diet (*H40*). Considering that no differences regarding protein and glycogen content were highlighted in the livers from fish fed the different experimental diets, the higher liver lipid content in fish from the *H40* group can be related to the different HM FA profile, known to be rich in MUFA and SFA rather than PUFAs [7,44].

## 5. Conclusions

Overall, the results obtained in the present study demonstrated that HM and PBM can be successfully used to replace Vp in a vegetable-based diet for gilthead seabream without negatively affecting fish growth performances and welfare. In addition, HM showed interesting effects in ameliorating the fish intestine condition, while PBM exerted beneficial effects on intestine nutrient absorption, suggesting that this ingredient can be used to counteract the negative side effects due to the high amount of plant ingredients in diets for gilthead seabream.

## Figures and Tables

**Table 1:** Ingredient (g 100 g^−1^) and proximate (% as fed) composition of the test diets.

Ingredient Composition	CV	H20	H40	P20	P40	H10P30	CF
Fish meals 1							54.0
Vegetable-protein mix 2	69	52.6	36.6	52.5	35.4	35.4	-
*Hermetia* meal 3	-	16.2	32.4	-	-	8.1	-
PBM 4	-	-	-	13.8	27.5	20.6	-
Feeding stimulants 5	5.5	5.5	5.5	5.5	5.5	5.5	5.5
Wheat meal *	0.4	1.6	4.5	3.0	5.6	5.5	3.0
Whole pea *	3.0	5.8	6.0	6.2	9.0	8.8	20.5
Fish oil 6	6.2	6.2	6.2	6.2	6.2	6.2	8.6
Veg. oil mix 7	11.4	8.4	5.4	9.8	8.2	7.4	6.5
Vit. & Min. Premix 8	0.3	0.3	0.3	0.3	0.3	0.3	0.3
Choline HCL	0.1	0.1	0.1	0.1	0.1	0.1	0.1
Sodium phosphate (NaH2PO4)	1.6	1.2	1.0	0.7	0.3	0.2	-
L-Lysine 9	0.5	0.2	0.2	0.1	0.1	0.1	-
DL-Methionine 10	0.5	0.4	0.3	0.3	0.3	0.3	-
Celite	1.5	1.5	1.5	1.5	1.5	1.5	1.5
**Proximate composition**							
Moisture	6.7	6.1	4.7	7.1	7.2	8.7	8.2
Protein (N × 6.25)	45.0	45.2	45.2	45.1	45.1	45.1	45.4
Total lipid	20.4	20.1	20.4	20.4	20.2	20.4	20.3
Ash	5.8	6.6	6.5	7.1	7.9	7.6	12.4
Chitin #	0.02	0.76	1.51	0.02	0.02	0.39	0.02

1, Fish meal mixture (% composition): 26% Pesquera Diamante Peru (66.3% crude ptotein (CP), 11.5% crude fat (CF); 74% Conresa 60, Conserveros Reunidos S.A. Spain (61.2% CP, 8.4% CF). 2, Vegetable-protein source mixture (% composition): dehulled, toasted soybean meal, 39; soy protein concentrate-Soycomil, 20; maize gluten, 18; wheat gluten, 15; rapeseed meal, 8. 3, ProteinX™, Protix, Dongen, The Netherlands (CP, 55.4%; CF, 20.8% as fed). 4, Poultry by-product meal from Azienda Agricola Tre Valli; Verona, Italy (CP, 65.6%; CF, 14.8% as fed). 5, Feeding stimulants g/100 diet fish protein concentrate: CPSP90- Sopropeche, France (82.6% CP), 3.5; Squid meal (80.3% CP), 2.0. 6, Fish oil: Sopropêche, France. 7, Vegetable oil mixture % composition: rapeseed oil, 56; linseed oil, 26; palm oil, 18. 8, Vitamin and mineral supplement (per kg of premix): Vit. A, 2,000,000 IU; Vit D3, 200,000 IU; Vit. E 30,000 mg; Vit. K3, 2500 mg; Vit.B1, 3000 mg; Vit. B2, 3000 mg: Vit B3, 20,000 mg; Vit. B5, 10,000 mg; Vit B6, 2000 mg, Vit. B9, 1500 mg; Vit. B12, 10 mg, Biotin, 300 mg; Stay C^®^, 90,000 mg; Inositol, 200,000 mg; Cu, 900 mg; Fe, 6000 mg; I, 400 mg; Se, 40 mg; Zn, 7500 mg. 9 L—lysine, 99%; Ajinomoto EUROLYSINE S.A.S; France. 10, DL—Methionine: 99%; EVONIK Nutrition & Care GmbH; Germany. * Wherever not specified, the ingredients composing the diets were obtained from local providers by Sparos Lda. # Estimated based on the chitin content of the ingredients used (squid meal, 0.9%, and *Hermetia illucens* meal, 4.69%).

**Table 2:** Semi-quantitative scoring system for the different parameters used as histopathological indexes of enteritis in gilthead seabream intestine.

	Score	Description
Mucosal folds fusion (MF f)	+	0–5 observation per section
	++	5–15 observation per section
	+++	>15 observation per section
Supranuclear vacuoles (SN v)	+	Scarce
	++	Diffused in the enterocytes
	+++	Abundantly filling enterocytes
Sub-mucosa width (SM w)	+	10–15 µm
	++	15–30 µm
	+++	>30 µm

**Table 3:** Oligonucleotide primers, annealing temperature (A.T.), and location (Gene Bank Accession Number) of each gene investigated in this study. hk: housekeeping genes.

Gene Name	Primer Sequence		A.T. (°C)	Gene Bank ID
	Forward	Reverse		
*Il1b*	ATCCAGCTGTCTTTCCCTCA	TTGCATGTCATCTCGGATTC	59	XM_030435228.1
*il10*	AACATCCTGGGCTTCTATCTG	TGTCCTCCGTCTCATCTG	60	JX976621.1
*tnfa*	CTGTGGAGGGAAGAATCGAG	CTTTCTGGTCCACCTCACCT	60	XM_030412624.1
*nfkb*	GTTTGTCGTGTCGTTGGGAG	CGAGTGGACAAGTGAGTGGA	58	XM_030403588.1
*myd88*	CCGTCGTCTGTGGCTAACAT	GTCCCACGCCTTTTTCAACC	56	XM_030399037.1
*tlr1*	CTTGTGCCCAGCAGTGTTTC	CGGTTTGTAGCACGGTCTTC	60	XM_030396315.1
*β-actin* (hk)	TCCTGCGGAATCCATGAGA	GACGTCGCACTTCATGATGCT	57	X89920.1
*rps18* (hk)	AGGGTGTTGGCAGACGTTAC	CTTCTGCCTGTTGAGGAACC	57	AM490061.1

**Table 4:** Growth performance, specific growth rate (SGR), relative feed intake (RFI), and feed conversion ratio (FCR) of gilthead sea bream kept at 23.4 ± 0.75 °C and fed the test diets over 12 weeks.

Dietary Treatments	Final Weight g/Fish	SGR	RFI g/kg ABW/d	FCR
CV	177.7 ± 2.10 ^c^	1.54 ± 0.01 ^c^	15.9 ± 0.21 ^b^	1.18 ± 0.05 ^b^
H20	187.5 ± 3.60 ^b^	1.59 ± 0.02 ^b^	15.8 ± 0.16 ^b^	1.13 ± 0.04 ^a^
H40	192.2 ± 0.80 ^a^	1.64 ± 0.02 ^a^	15.6 ± 0.07 ^b^	1.11 ± 0.04 ^a^
P20	191.6 ± 1.47 ^a^	1.63 ± 0.01 ^a^	15.9 ± 0.33 ^b^	1.11 ± 0.03 ^a^
P40	192.3 ± 0.36 ^a^	1.63 ± 0.01 ^a^	15.8 ± 0.38 ^b^	1.10 ± 0.02 ^a^
H10P30	190.7 ± 1.32 ^ab^	1.62 ± 0.01 ^a^	15.7 ± 0.08 ^b^	1.11 ± 0.02 ^a^
CF	180.1 ± 1.41 ^c^	1.55 ± 0.02 ^c^	16.4 ± 0.08 ^a^	1.21 ± 0.02 ^b^
**± esm**	2.27	0.017	0.22	0.022

Mean ± SD for each dietary group. Different superscript letters represent significantly different values (*p* < 0.05).

**Table 5:** Mucosal fold morphometric evaluation and histological index scores in the medium (**a**) and distal intestine (**b**) from fish fed experimental diets.

**Groups**	MF	MF f	SN v	SM w	Groups	MF	MF f	SN v	SM w
	**(µm)**					**(µm)**			
**CV**	853 ± 12 ^b^	+++	+	++	**CV**	631 ± 79 ^b^	+++	+++	+++
**H20**	1003 ± 88 ^a^	+	+	+	**H20**	672 ± 97 ^b^	+	++	+
**H40**	1092 ± 88 ^a^	+	++	+	**H40**	813 ± 15 ^a^	+	+	+
**P20**	1010 ± 12 ^a^	+	++	+	**P20**	965 ± 94 ^a^	+	+	+
**P40**	1023 ± 11 ^a^	+	++	+	**P40**	846 ± 59 ^a^	+	+	+
**H10P30**	1073 ± 23 ^a^	+	+++	+	**H10P30**	948 ± 95 ^a^	+	+	+
**CF**	1100 ± 13 ^a^	+	+++	+	**CF**	821 ± 80 ^a^	+	+	+
		**(a)**					**(b)**		

MF: mucosal fold height; MF f: mucosal fold fusion; SN v: supranuclear vacuoles; SM w: submucosa width. Folds height is expressed by the means of the measurements performed ± SD. Different superscript letters indicate significant differences among the experimental groups (a,b: *p* < 0.05).

**Table 6:** Histological evaluation of the percentage of fat fraction (PFF) in liver histological sections. Data are reported as mean ± SD. No significant differences were detected among groups.

Diets	PFF (%)
CV	58.8 ± 1.8
H20	63.5 ± 2.2
H40	65.6 ± 2.2
P20	61.7 ± 1.3
P40	63.1 ± 2.3
H10P30	62.9 ± 2.3
CF	62.4 ± 1.4

## Data Availability

Not available.

## Supplementary Materials

The following are available online at https://www.mdpi.com/2076-2615/11/3/677/s1: Table S1: Proximate analysis (% as fed) and fatty acid profile (% Fames) of commercial insect meal (HM) and poultry by-product meal (PBM) used as test ingredients in the experiment.
