# Supplementary material for: Hermetia illucens and Poultry by-Product Meals as Alternatives to Plant Protein Sources in Gilthead Seabream (Sparus aurata) Diet: A Multidisciplinary Study on Fish Gut Status
Source: Animals (Basel). 2021 Mar 4;11(3):677. doi: 10.3390/ani11030677 (PMC8001786; doi:10.3390/ani11030677)
Supplement: Supplementary file 1 [file animals-11-00677-s001.pdf]

animals

Supplementary Materials: *Hermetia illucens* and Poultry by-Product Meals as Alternatives to Plant Protein Sources in Gilt-head Seabream (*Sparus aurata*) Diet. A multidisciplinary Study on Fish Gut Status

Basilio Randazzo, Matteo Zarantoniello, Gloriana Cardinaletti, Roberto Cerri, Elisabetta Giorgini, Alessia Belloni, Michela Contò, Emilio Tibaldi and Ike Olivotto

Table S1. Proximate analysis (% as fed) and fatty acid profile (% Fames) of commercial insect meal (HM) and poultry by-product meal (PBM) used as test ingredients in the experiment.

Proximate Composition	HM	PBM
Moisture	4.42	5.80
N × 6.25	53.10	65.56
Ash	6.37	12.41
Chitin[#]	4.69	-
Crude lipid	20.80	14.83
**Fatty acids**		
SFA	61.08	30.70
C12	31.82	0.06
C14	7.09	0.93
C18	3.30	7.73
MUFA	18.8	48.7
C16:1	2.3	5.6
C18:1c	13.5	38.8
PUFA	20.2	20.6
PUFA n6	19.1	19.6
C18:2n6	18.8	17.3
C20:4n6	0.2	1.4
PUFA n3	1.0	1.0
C18:3n3	0.9	0.1
C20:5n3 EPA	-	0.1
C22:6n3DHA	0.1	0.1
n6/n3	18.9	19.2
